# 699. Clinical Outcomes and Management of NAAT-Positive/Toxin-Negative *Clostridium difficile* Infection: A Systematic Review and Meta-Analysis

**DOI:** 10.1093/ofid/ofad500.761

**Published:** 2023-11-27

**Authors:** Connor Prosty, Ryan Hanula, Khaled Katergi, Yves Longtin, Emily McDonald, Todd C Lee

**Affiliations:** McGill University, Montréal, Quebec, Canada; McGill, Montreal, Quebec, Canada; Université de Montreal, Montreal, Quebec, Canada; Jewish General Hospital, Montreal, Montreal, QC, Canada; McGill University Health Centre, Montreal, Quebec, Canada; McGill University, Montréal, Quebec, Canada

## Abstract

**Background:**

Standalone nucleic acid amplification tests (NAATs) are increasingly used to diagnose *Clostridium difficile* infections (CDI), although they may be unable to distinguish colonization from disease. A two-stage algorithm pairing NAAT with a toxin immunoassay (Toxin) has been proposed to improve specificity. We sought to evaluate clinical outcomes among patients testing NAAT+/Toxin+ vs NAAT+/Toxin- by conducting a systematic review and meta-analysis. We further compared outcomes between NAAT+/Toxin- patients who were and were not treated.

**Methods:**

We searched EMBASE and MEDLINE from inception to September 17, 2022 for articles comparing CDI outcomes among symptomatic patients tested by NAAT and Toxin tests. The risk differences (RD) of all-cause mortality and CDI recurrence were computed by random effects meta-analysis between NAAT+/Toxin+ and NAAT+/Toxin- patients, as well as between treated and untreated NAAT+/Toxin- patients.

**Results:**

Twenty-three observational studies comprising 11749 patients were included. 30-Day all-cause mortality was not significantly different between NAAT+/Toxin+ and NAAT+/Toxin- patients (8.4% vs 6.8%, respectively; RD=0.2%, 95%CI -2.4, 2.9; I^2^=52.9%). Recurrence at 60 days was significantly higher among NAAT+/Toxin+ (19.8%) vs NAAT+/Toxin- patients (11.0%) (RD=7.7%, 95%CI=4.6, 10.7, I^2^=37.4%). Among treated compared to untreated NAAT+/Toxin- patients, the all-cause 30-day mortalities were 5.0% and 14.9%, respectively (RD=-9.5%, 95%CI=-15.0, -3.9, I^2^=0.0%), but 60-day recurrence was not significantly different (11.6% vs 7.0%, respectively; RD=5.3%, 95%CI -1.7, 12.2; I^2^=47.0%).

**Conclusion:**

Compared to NAAT+/Toxin- patients, NAAT+/Toxin+ patients had a greater risk of recurrence at 60 days, but not all-cause mortality. Treatment of NAAT+/Toxin- patients was associated with reduced all-cause mortality, but not recurrence. While subject to the limitations of observational studies including the potential for confounding by indication and immortal time bias, these results suggest that treatment of NAAT+/Toxin- patients may be beneficial. A strategy comparing standalone NAAT or staged testing with up front combined NAAT/Toxin testing could be the topic of a randomized controlled trial to determine cost and efficacy.

**Disclosures:**

**All Authors**: No reported disclosures

